# Reports on the Hospitals of the United Kingdom

**Published:** 1912-02-24

**Authors:** Henry Burdett


### REPORTS ON THE

### Hospitals of the United Kingdom.

By SIR HENRY BURDETT, K.C.B., K.C.V.O.

### LXIII.—Sunderland and Durham County Eye Infirmary.

SEVENTY-FIVE PER CENT. OF THE EXPENDITURE PROVIDED BY THE WORKING CLASSES.

This is an ancient foundation, established in 1836 and removed to its present site in the Stockton Road in 1893. We decided to inspect it because it contains an unusually large number of beds for an institution of its kind, and we were agreeably surprised to find how efficient it proved to be in all respects except the quarters for the resident and nursing staff. From the patients' and the scientific point of view, everything throughout the hospital was satisfactory. Indeed, we formed the impression from the quality and amount of the actual work done each year that this hospital must be an invaluable asset to the earning power of the working classes of Sunderland and the district. It is pleasant to find from the annual report that the workmen have emphatically endorsed this opinion by their annual contribution to the receipts and revenue of the institution. The workmen's contributions in fact amounted in 1910 to upwards of three-fourths of the total expenditure.

### Matters of Detail.

It is pleasant, therefore, to be able to record that the hospital is well kept and ordered, that the recent additions to the out-patient department include a most excellent dark-room with modern appliances, an *x*-ray and electrical department equipped with all useful electrical apparatus which modern science has placed at the disposal of the medical profession, and in addition a Stevens' photometer, a Worth-Black electric amblyoscope, an electrically lighted Maddox tangent scale, an electric Morton-Marple ophthalmoscope, a Wurdeman's transilluminator, two additional Morton ophthalmoscopes, and an illuminated testing cabinet. This department is fitted with a powerful induction coil and all the necessary apparatus for detecting and localising a foreign body in the eye. There is also a switch-board for galvanisation, faradisation and ionic medication, and for electrolysis of misplaced eyelashes. A galvano-cautery and various lamps for transillumination are also provided. An operation theatre has also been added within the year, which has been brought up to date with modern fittings, including a new operating-table, Haab's giant magnet, small hand-magnets, etc., and is well supplied with every instrument necessary to ophthalmic surgery. The window is composed of a single large sheet of plate-glass facing north, which obviates the reflection of cross-bars on the cornea during operations. At night the light is supplied by a 300-c.p. electric lamp. An expenditure of upwards of £1,000 has been made to bring the institution up to date, and most of the instruments and appliances are new within the last two years. The out-patient waiting-hall, an older erection, is, however, in need of a more adequate system of ventilation. Each of the children's wards, in our opinion, contains at least one cot too many for the floor, cubic and superficial space available.

### An Unaccountable Omission.

The whole hospital contains evidence of the keen interest taken in its progressive development by the General Committee, which makes it the more astonishing to find that in fact practically no accommodation has been provided for the house surgeon, who has always been a gentleman of the highest qualifications on whom much responsibility rests; nor for the nurses, most of whom have at present to sleep out. In these circumstances we ventured to make the following entry in the visitors' book on September 19, 1911:—

"I came to inspect this hospital on account of its size, knowing nothing else about it. I have been impressed with the excellence of the arrangements for the treatment of the patients, and by their efficiency. They are very encouraging and exceptionally good and up to date. The work, therefore, is evidently proceeding satisfactorily, and the evidence of the high standard which animates the workers of all grades is plain and gratifying to see. In such circumstances, seeing that funds are adequate, it is a matter for regret and surprise that the comforts of the staff, and especially of the nurses, should have been wholly disregarded. I venture to record my opinion that in no other hospital where the staff do their duty to an equal extent is it possible to find the accommodation for the residents unprovided as at this institution.

"It is essential that this grave reflection upon the Board of Management should be promptly removed by having plans prepared, and the buildings so reconstructed as to provide full sleeping and day accommodation for the nursing staff, and preferably for the resident medical officer too. Until such reconstruction is properly carried out, this hospital, excellent as it is from the scientific and the patients' point of view, must remain the most striking example amongst British hospitals of the forgetfulness (through a want of apprehension, no doubt) which may dominate an otherwise efficient Committee for years and years.—(Signed) Henry C. Burdett."

We hoped that this protest might prove effective by securing that an additional story should be promptly added to the present buildings, seeing that the institution is in the financial position of having ample funds and most generous and interested contributors in the workmen of Sunderland and the district, to whom this hospital is of inestimable value.

The above entry caused the Committee to take immediate action, which resulted in the provision of a comfortable sitting-room for the nurses' use, though it entailed two day-nurses and one night-nurse sleeping out, a most undesirable arrangement. We came to the conclusion it would be easiest and best to raise the buildings another story, but from the latest reports we have received it would appear the Committee had under consideration at the end of the year a proposal for acquiring further property on which they could build adequate accommodation for the staff of the institution. We are not aware whether the proposal has been adopted and put into force or not.

The Board-room.

We have referred to the board-room before in the course of these reports, and the more hospitals we inspect the more we are inclined to recognise that the atmosphere and condition of the board-room is often an invaluable index to the probable condition of the whole establishment, and to the presence or absence of efficient control. The board-room of this hospital is a simple, well-proportioned chamber, plainly furnished with chairs that are comfortable, whilst being of the simple, businesslike kind which ought to characterise the furniture of a well-administered office. Every committeeman has a chair of equal comfort to that provided for the chairman, but there is an absence of all attempt at luxury, and an evident desire, whilst providing comfortable seats, to keep in mind that economy in the board-room may mean the enforcement of at least equal economy throughout the institution to which it may be attached. The working classes have done their best, and nobly too, in support of this hospital which has a supreme value to them.

